# Reevaluating the Necessity of Performing Routine Laboratory Tests in the First Week After Joint Replacement Surgery

**DOI:** 10.7759/cureus.107367

**Published:** 2026-04-19

**Authors:** Yoshiaki Miyake, Toru Takagi

**Affiliations:** 1 Orthopedic Surgery, Japanese Red Cross Okayama Hospital, Okayama, JPN

**Keywords:** allogeneic blood transfusion, electrolyte abnormality, joint replacement surgery, laboratory test, postoperative management

## Abstract

Purpose

Routine postoperative laboratory tests are commonly included in clinical paths following joint replacement surgery in Japan. However, with the global trend toward outpatient arthroplasty, the necessity of frequent testing is being questioned. Therefore, this study aimed to evaluate the necessity of routine and frequent laboratory tests during the first postoperative week of patients undergoing joint surgeries.

Methods

This retrospective study included 201 patients who underwent primary arthroplasty (64 total hip arthroplasty (THA), 85 total knee arthroplasty, and 52 unicompartmental knee arthroplasty) between April 2022 and September 2023. Laboratory tests were performed preoperatively and on postoperative days 1, 3, and 7. Abnormalities in hematologic, renal, hepatic, and electrolyte parameters and whether these abnormalities led to therapeutic interventions were examined.

Results

Abnormal laboratory values were common, including low hemoglobin levels (79%), elevated C-reactive protein levels (100%), and mild electrolyte or hepatic enzyme changes. However, therapeutic interventions based on these tests were required in only eight (4%) patients. Two patients who underwent THA received allogeneic transfusion, one was treated for hypokalemia and four for hyperkalemia, and one discontinued edoxaban due to suspected liver dysfunction. No interventions were executed based on day 3 laboratory findings.

Conclusions

Although abnormal laboratory results were frequent after joint replacement surgery, they rarely led to therapeutic intervention. Most abnormalities were clinically insignificant or transient. Routine testing on postoperative day 3 may be unnecessary in well-managed patients, and omitting it could reduce patient burden and healthcare costs without compromising safety. Careful monitoring with preoperative and immediate postoperative tests may be sufficient.

## Introduction

Joint replacement surgery is highly effective in relieving pain and restoring function in patients with joint diseases. The number of joint surgeries performed annually is increasing [1]. The increasing popularity of minimally invasive surgery [2,3], in which muscles are spared, has enabled patients to leave the hospital and return to normal life earlier after surgery [4,5]. However, many hospitals in Japan still require approximately two weeks of inpatient care after joint replacement surgery. In general, perioperative management using a clinical pathway is useful for early discharge [6,7]. Laboratory tests are often performed routinely after joint replacement surgery according to the clinical pathway, and in Japan, many clinical pathways specify three laboratory tests during the first postoperative week [8]. However, outpatient joint replacement is becoming more common worldwide [9,10], and routine and frequent postoperative laboratory tests are rarely performed in such cases. Frequent laboratory tests are burdensome for the patients themselves, nurses who take blood samples, and medical laboratory technologists who perform examinations. It also poses an economic healthcare problem if examinations are unnecessary. However, only a few studies have examined the necessity of postoperative laboratory tests [11,12], and these are not from Japan. Therefore, this study aimed to evaluate the clinical necessity of routine and frequent laboratory testing during the first postoperative week in patients undergoing joint replacement surgery. The primary outcome of this study was the proportion of patients in whom abnormal laboratory test results led to therapeutic intervention. In particular, we aimed to determine whether routine laboratory testing on postoperative day 3 provides actionable clinical value in patients undergoing primary total hip arthroplasty (THA), total knee arthroplasty (TKA), or unicompartmental knee arthroplasty (UKA).

## Materials and methods

This retrospective comparative study was conducted in accordance with the principles of the Declaration of Helsinki and was retrospectively approved by the Ethics Committee of Japanese Red Cross Okayama Hospital (approval number: 2025-71; approval date: December 12, 2025). The requirement for informed consent was waived owing to the retrospective nature of the study.

The study included 201 patients who had undergone initial arthroplasty between April 2022 and September 2023 at our institution, including 48 male patients and 153 female patients, with a mean age at surgery of 71.9 (±10.0) years.

Inclusion and exclusion criteria

The inclusion criteria were as follows: (i) Patients who underwent primary THA, TKA, or UKA; (ii) Elective surgery performed at our institution during the study period. The exclusion criteria were as follows: (i) Patients who underwent urgent surgery for femoral neck fractures; (ii) Revision arthroplasty for loosening or infection.

Of these, 64, 85, and 52 patients underwent THA, TKA, and medial UKA, respectively.

Laboratory tests were performed on these patients preoperatively (within one month before surgery) and before breakfast on postoperative days 1, 3, and 7 to measure white blood cell (WBC) and platelet counts and hemoglobin, blood urea nitrogen (BUN), creatinine, serum sodium (Na), serum potassium (K), aspartate aminotransferase (AST), alanine aminotransferase (ALT), γ-glutamyltransferase (γ-GTP), total bilirubin (T-Bil), and C-reactive protein (CRP) levels. The presence or absence of abnormal laboratory values and whether these abnormalities led to therapeutic intervention were examined. Abnormal values were defined as follows, based on the institutional reference ranges: WBC count >8,600/µL (reference range: 3,300-8,600/µL), hemoglobin level <13.7 g/dL in males and <11.6 g/dL in females (reference range: males, 13.7-17.5 g/dL; females, 11.6-14.8 g/dL), platelet count <158,000/µL (×10³/µL; reference range: 158-348 ×10³/µL), Na level <138 or >145 mmol/L (reference range: 138-145 mmol/L), K level <3.6 or >4.8 mmol/L (reference range: 3.6-4.8 mmol/L), AST level >30 U/L (reference range: 13-30 U/L), ALT level >42 U/L in males and >23 U/L in females (reference range: males, 10-42 U/L; females 13-30 U/L), γ-GTP level >64 U/L in males and >32 U/L in females (reference range: males, 13-64 U/L; females 9-32 U/L), T-Bil level >1.5 mg/dL (reference range: 0.4-1.5 mg/dL), and CRP level >0.14 mg/dL (reference range: 0.00-0.14 mg/dL).

In our department, patients aged <80 years who underwent THA with a preoperative hemoglobin level of ≥11.0 g/dL are required to donate four units of autologous blood, and the stored autologous blood is returned postoperatively regardless of the amount of perioperative blood loss or presence of postoperative anemia. Preoperative autologous blood donation was not performed in patients undergoing knee arthroplasty. Cefazolin is used as a postoperative antibiotic and is administered within 48 h after surgery regardless of postoperative inflammatory laboratory findings. Postoperative analgesics include nonsteroidal anti-inflammatory drugs (NSAIDs) or acetaminophen, depending on renal function. Edoxaban is started on the second postoperative day depending on wound condition and renal function and continued for 10 days to prevent venous thromboembolism. Therapeutic interventions were not initiated based on laboratory abnormalities alone but were generally considered when abnormal values were accompanied by clinical symptoms (e.g., lightheadedness, fatigue), significant deviation from reference ranges, or when the treating surgeon judged that medical treatment was necessary based on the patient's overall clinical condition.

No formal sample size calculation was performed because this was a retrospective observational study including all eligible patients during the study period. This study was descriptive in nature, and no inferential statistical comparisons were performed. Descriptive statistical analyses were performed using IBM SPSS Statistics for Windows, Version 23 (Released 2015; IBM Corp., Armonk, New York, United States).

## Results

The percentages of patients with abnormal laboratory test results before and after surgery (postoperative days 1, 3, and 7) are summarized in Table 1.

**Table 1:** Proportion of patients with abnormal laboratory test results before and after joint replacement surgery. Abnormal laboratory values were assessed preoperatively and on postoperative days 1, 3, and 7. Values are presented as the percentage of patients with at least one abnormal result at each time point. Abnormalities were defined according to the institutional reference ranges. Preop, preoperative; Postop, postoperative; WBC, white blood cell count; BUN, blood urea nitrogen; Na, sodium; K, potassium; AST, aspartate aminotransferase; ALT, alanine aminotransferase; γ-GTP, gamma-glutamyltransferase; T-Bil, total bilirubin; CRP, C-reactive protein.

	Preop	Postop day 1	Postop day 3	Postop day 7
WBC	11%	58%	39%	16%
Hb	26%	70%	71%	70%
Plt	2%	8%	11%	8%
BUN	25%	15%	10%	39%
Creatinine	24%	17%	14%	26%
Low Na	2%	7%	2%	4%
High Na	3%	0%	0%	1%
Low K	2%	5%	10%	3%
High K	10%	1%	1%	10%
AST	10%	18%	14%	19%
ALT	18%	8%	7%	29%
γ-GTP	21%	16%	21%	33%
T-Bil	1%	4%	1%	2%
CRP	45%	100%	99%	95%

At least one abnormal postoperative laboratory value was observed in a substantial proportion of patients. Specifically, abnormal values were observed for WBC count in 124 patients (62%), hemoglobin level in 158 patients (79%), platelet count in 28 patients (14%), BUN level in 83 patients (41%), creatinine level in 53 patients (26%), Na level in 23 patients (11%), K level in 49 patients (24%), AST level in 70 patients (35%), ALT level in 72 patients (36%), γ-GTP level in 69 patients (34%), T-Bil level in 10 patients (5%), and CRP level in all patients (201/201, 100%).

Among patients with abnormal Na levels, hyponatremia was observed in 20 patients (10%) and hypernatremia in three patients (1%). Among patients with abnormal K levels, hypokalemia was observed in 26 patients (13%) and hyperkalemia in 23 patients (11%). Hyponatremia and hypokalemia were more common in the early postoperative period, whereas hyperkalemia tended to increase as the postoperative course progressed.

Despite the high frequency of laboratory abnormalities, therapeutic intervention based on postoperative laboratory findings was required in only eight patients (4.0%, 8/201). Details of therapeutic interventions and associated adverse events are summarized in Table 2.

**Table 2:** Postoperative laboratory abnormalities requiring therapeutic intervention. This table summarizes cases in which abnormal postoperative laboratory findings resulted in therapeutic intervention during the first postoperative week. Data include the type of arthroplasty, postoperative day of detection, laboratory abnormality, and corresponding treatment. THA, total hip arthroplasty; TKA, total knee arthroplasty; Hb, hemoglobin; K, potassium; AST, aspartate transaminase; ALT, alanine transaminase; γ-GTP, γ-glutamyltransferase

Patient number	Sex	Age (years)	Arthroplasty	Postoperative day of detection	Abnormal values	Treatment
1	Female	81	THA	1	Hb 7.3 g/dL	Allogeneic blood transfusion
2	Female	85	THA	1	Hb 6.9 g/dL	Allogeneic blood transfusion
3	Female	79	TKA	1	K 2.8 mmol/L	Potassium asparagine
4	Female	63	THA	7	K 5.5 mmol/L	Calcium polystyrene sulfonate
5	Female	78	TKA	7	K 5.6 mmol/L	Calcium polystyrene sulfonate
6	Female	91	TKA	7	K 5.4 mmol/L	Calcium polystyrene sulfonate
7	Female	86	TKA	7	K 6.1 mmol/L	Calcium polystyrene sulfonate
8	Male	65	TKA	7	AST 134 U/L, ALT 179 U/L, γ-GTP 636 U/L	Discontinued edoxaban

Of these eight patients, two patients aged over 80 years who underwent THA and did not undergo preoperative autologous blood donation required allogeneic blood transfusion on postoperative day 1 because of low hemoglobin levels. One patient with hypokalemia on postoperative day 1 was treated with oral potassium asparagine. Four patients with hyperkalemia on postoperative day 7 were treated with oral calcium polystyrene sulfonate. In one patient, elevated liver enzyme levels (AST, ALT, and γ-GTP) on postoperative day 7 were attributed to edoxaban, and the medication was discontinued.

Of the eight patients who underwent therapeutic intervention, two patients with anemia reported mild lightheadedness, whereas the remaining six patients were asymptomatic. The four patients with hyperkalemia showed no electrocardiographic abnormalities. No therapeutic interventions were required based on laboratory findings on postoperative day 3.

**Figure 1:**
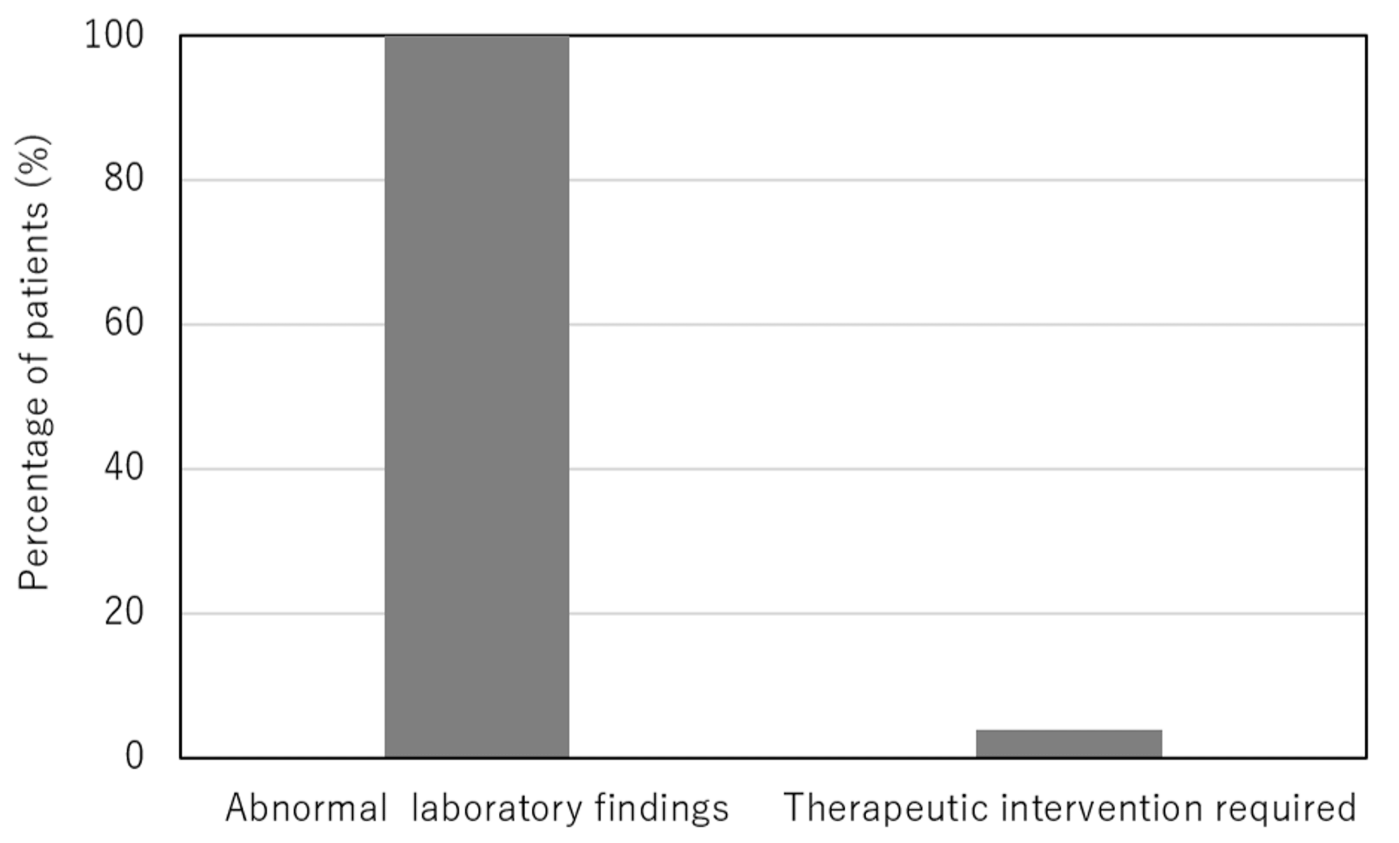
Discrepancy between postoperative laboratory abnormalities and required therapeutic interventions. Bar graph illustrating the high prevalence of abnormal postoperative laboratory findings compared with the low proportion of patients who required therapeutic intervention following joint replacement surgery.

Figure 1 illustrates the marked discrepancy between the high frequency of abnormal postoperative laboratory findings and the low rate of therapeutic interventions required.

## Discussion

This study reflects real-world perioperative management in Japanese general hospitals, where inpatient care is longer and routine laboratory testing is commonly incorporated into clinical pathways. Despite the widespread use of this practice, postoperative laboratory blood tests led to therapeutic intervention in only 4% of the cases, with no serious complications observed.

The "10/30" rule (hemoglobin <10 g/dL and hematocrit <30%) is the classic indication for allogeneic blood transfusion [13], and a high rate (10-20%) of allogenic blood transfusion after primary joint arthroplasty has been previously reported [14,15]. However, the 2015 American Society of Anesthesiologists guidelines state that allogeneic blood transfusion is not necessary for a hemoglobin level of ≥10 g/dL, transfusion is desirable at ≤6 g/dL, and the requirement for 6-10 g/dL hemoglobin should be determined based on persistent bleeding, circulating plasma volume, cardiovascular reserve, and response to anemia [16]. They suggest that observation is sufficient if there are no clinical symptoms at a hemoglobin level of ≥8 g/dl. In this study, allogeneic blood transfusions were required in only two (1%) patients who underwent THA, aged >80 years, and had no autologous blood stock due to preoperative anemia.

In the past, the duration of postoperative antibiotic administration may have been extended based on the results of inflammatory reactions on laboratory tests on the first and third postoperative days. However, recently, antimicrobial prophylaxis for >24-48 h after surgery has not been recommended to prevent bacterial resistance [17,18]. Prolonged administration of prophylactic antimicrobial agents is not only ineffective in preventing infection but also increases the rate of resistance of indigenous skin bacteria and is considered harmful. Postoperative CRP levels increase owing to a physiological inflammatory response and reach the peak 2-3 days after surgery. Since high CRP values can be observed even in the absence of infection and may not increase even when there is an infection, it is considered that a high CRP value within one week after surgery is not evidence of infection [19,20]. In this study, all patients had high postoperative CRP levels; however, antimicrobial prophylaxis was completed within 48 h after surgery, and surgical site infection was not observed in any patient.

Hormones reduce urine output during the acute postoperative period to prevent fluid loss. Aldosterone causes Na and H2O to be reabsorbed, and K to be eliminated. Vasopressin also causes the reabsorption of Na and H2O. Hyponatremia and hypokalemia are common after orthopedic surgery and are reported in 13.2% and 53.1% of patients, respectively, after arthroplasty [21,22]; the severity of hyponatremia is considered low. In the present study, hyponatremia and hypokalemia occurred in only 10% and 13% of the patients, respectively, during the first postoperative week. None of the patients had subjective symptoms, and only one patient (K 2.8 mmol/L), who did not have subjective symptoms, was treated with oral asparagine, after which hypokalemia improved. In addition, hyperkalemia after joint replacement surgery is a rare problem, with mild to moderate hyperkalemia reported in 2.6% of cases [23]. In this study, four patients (2%) were orally administered calcium polystyrene sulfonate. However, according to the Renal Association (UK Kidney Association) Clinical Practice Guideline [24], the treatment criteria for acute hyperkalemia are a potassium level of ≥7.0 mmol/L or ≥6.0 mmol/L with accompanying electrocardiographic abnormalities. Therefore, in this study, all four patients might have been adequately managed with observation alone without medication.

Postoperative liver dysfunction can be drug induced, post-transfusion, or due to circulatory disturbances. Postoperative liver dysfunction after joint replacement surgery is rare, and no reports specific to liver dysfunction after joint replacement surgery have been published. In this study, allogeneic blood transfusions were required in only two patients, and none of them experienced massive bleeding that caused hepatic dysfunction. Temporary mild liver dysfunction was observed in approximately 30-40% of patients. Only one patient (0.5%) was considered to have drug-induced liver dysfunction, in whom liver function improved spontaneously two weeks postoperatively after discontinuing edoxaban, which was considered to be the cause. No patients with severe hepatic dysfunction required therapeutic intervention.

Renal dysfunction is generally classified as prerenal (inadequate renal perfusion), renal (acute tubular necrosis, acute glomerulonephritis, and nephrotoxic substances), and postrenal (obstruction of the urinary system). Postoperative renal dysfunction is considered prerenal owing to inadequate renal perfusion and nephrotoxic substances. The incidence of acute renal dysfunction after joint replacement surgery ranges from 5 to 10% [25]. In the present study, no cases of renal dysfunction requiring treatment were observed. Among drugs used in the perioperative period, antibiotics and analgesics are associated with postoperative renal dysfunction. The 2016 Japanese guidelines for the treatment of drug-induced kidney injury [26] state that the use of NSAIDs for postoperative pain in patients with normal renal function does not result in a clinically significant decrease in renal function, although a significant decrease in creatinine clearance is observed immediately postoperatively. In this study, postoperative antibiotics were discontinued within 48 h, and acetaminophen was administered for postoperative analgesia in patients with preoperative renal dysfunction. Therefore, none of the patients developed significant renal dysfunction, and none required discontinuation of NSAIDs due to renal dysfunction.

From a cost-effectiveness perspective, selective postoperative laboratory testing based on clinical symptoms and risk factors may reduce unnecessary healthcare expenditure, patient discomfort associated with repeated blood sampling, and staff workload related to ordering and reviewing laboratory results. While routine testing may facilitate early detection of asymptomatic abnormalities such as anemia or electrolyte imbalance, our findings suggest that frequent laboratory testing does not necessarily translate into increased therapeutic interventions in uncomplicated primary arthroplasty patients. Therefore, a more individualized approach to postoperative laboratory monitoring may improve resource utilization without compromising patient safety.

This study has several limitations. First, the sample size was relatively small, and this was a retrospective, single-center study, which may limit the generalizability of the findings. Second, there were no predefined criteria for therapeutic interventions, and treatment decisions were made at the discretion of the attending surgeons based on the overall clinical condition of each patient. Third, semi-emergency surgeries, such as femoral neck fractures, and highly invasive revision surgeries were excluded; therefore, the findings may not be applicable to these patient populations. Furthermore, perioperative management protocols and clinical decision-making processes may differ across institutions, which may limit the external generalizability of our findings.

In the present study, no serious complications occurred, and no patients required therapeutic intervention based on laboratory test results obtained on postoperative day 3. These findings suggest that careful clinical follow-up based on preoperative and postoperative day 1 laboratory results might be sufficient, and that routine laboratory testing on postoperative day 3 may be unnecessary in patients undergoing uncomplicated primary joint arthroplasty.

## Conclusions

In uncomplicated patients undergoing primary THA, TKA, or UKA under standardized perioperative management, routine laboratory testing on postoperative day 3 may have limited clinical value. Our findings suggest that selective postoperative laboratory testing based on patient symptoms and clinical risk factors, rather than routine testing for all patients, may be a more efficient strategy for postoperative monitoring.
